# Evidence of adaptation, niche separation and microevolution within the genus *Polaromonas* on Arctic and Antarctic glacial surfaces

**DOI:** 10.1007/s00792-016-0831-0

**Published:** 2016-04-20

**Authors:** Jan Gawor, Jakub Grzesiak, Joanna Sasin-Kurowska, Piotr Borsuk, Robert Gromadka, Dorota Górniak, Aleksander Świątecki, Tamara Aleksandrzak-Piekarczyk, Marek K. Zdanowski

**Affiliations:** Laboratory of DNA Sequencing and Oligonucleotide Synthesis, Institute of Biochemistry and Biophysics, Polish Academy of Sciences, Pawińskiego 5a, 02-106 Warsaw, Poland; Department of Antarctic Biology, Institute of Biochemistry and Biophysics, Polish Academy of Sciences, Pawińskiego 5a, 02-106 Warsaw, Poland; Institute of Genetics and Biotechnology, Faculty of Biology, Warsaw University, Pawińskiego 5a, 02-106 Warsaw, Poland; Department of Microbiology, Faculty of Biology and Biotechnology, University of Warmia and Mazury, Oczapowskiego 1A, 10-719 Olsztyn, Poland; Department of Microbial Biochemistry, Institute of Biochemistry and Biophysics, Polish Academy of Sciences, Pawińskiego 5a, 02-106 Warsaw, Poland

**Keywords:** *Polaromonas*, 16S rRNA gene, ITS, Glacier, Bacteria, Biogeography

## Abstract

*Polaromonas* is one of the most abundant genera found on glacier surfaces, yet its ecology remains poorly described. Investigations made to date point towards a uniform distribution of *Polaromonas* phylotypes across the globe. We compared 43 *Polaromonas* isolates obtained from surfaces of Arctic and Antarctic glaciers to address this issue. 16S rRNA gene sequences, intergenic transcribed spacers (ITS) and metabolic fingerprinting showed great differences between hemispheres but also between neighboring glaciers. Phylogenetic distance between Arctic and Antarctic isolates indicated separate species. The Arctic group clustered similarly, when constructing dendrograms based on 16S rRNA gene and ITS sequences, as well as metabolic traits. The Antarctic strains, although almost identical considering 16S rRNA genes, diverged into 2 groups based on the ITS sequences and metabolic traits, suggesting recent niche separation. Certain phenotypic traits pointed towards cell adaptation to specific conditions on a particular glacier, like varying pH levels. Collected data suggest, that seeding of glacial surfaces with *Polaromonas* cells transported by various means, is of greater efficiency on local than global scales. Selection mechanisms present of glacial surfaces reduce the deposited *Polaromonas* diversity, causing subsequent adaptation to prevailing environmental conditions. Furthermore, interactions with other supraglacial microbiota, like algae cells may drive postselectional niche separation and microevolution within the *Polaromonas* genus.

**Electronic supplementary material:**

The online version of this article (doi:10.1007/s00792-016-0831-0) contains supplementary material, which is available to authorized users.

## Introduction

Biogeography has always been a topic of major interest for the scientific community (Hubbell 2001). It was mainly focused on spatial distribution of multicellular organisms, neglecting the microbiome, including prokaryotes (Nemergut et al. 2011; Ramette and Tiedje 2007). This was mainly due to the tremendous dispersal potential of microbes, and only environmental selection determining which species were abundant (Martiny et al. 2006). Indeed at the genus level, many prokaryotes have a cosmopolitan distribution in the environment at local, regional, and continental scales (Rodrigues et al. 2009). However, further investigations of genetic, phenotypic and physiological microbial features revealed profound differences between isolates obtained from geographically distant areas, showing that endemism was more common than previously thought. These findings best fitted well-isolated extreme habitats, where the inhospitable outside environment hindered the dispersal of highly specialized microbes, leading to geographic isolation and subsequent neutral divergence (Ramette and Tiedje 2007).

In many ways, glaciers may be seen as island-like habitats. Those extreme ecosystems are featured on every continent and separated by large expanses of temperate terrestrial and marine areas (Shiklomanov 1993; Paterson 1994). Although not entirely isolated, (connected to some extent through the upper atmosphere via the movement of cold air masses) they are an environment where profound selective forces are at work (Hodson et al. 2008).

The bacterial genus *Polaromonas* seems to be among the dominant bacterial taxa in glacial ice since closely aligned sequences were discovered by metagenomic studies of glacial habitats worldwide, making *Polaromonas* one of the model taxons for investigating microbial distribution patterns in the terrestrial cryosphere (Willems 2014).

Papers regarding this topic were published previously (Darcy et al. 2011), using culture independent approach based on 16S rRNA gene sequences. Employment of large data sets to investigate genetic dispersal of *Polaromonas* phylotypes across global scales has led to the conclusion of a very weak genetic isolation between glacier habitats.

As much as environmental small subunit ribosomal gene analysis has advanced our understanding of microbial communities, it has its limitations due to persistence of extracellular DNA released into environment (Nielsen et al. 2007). Culture obtained bacterial isolates still can provide a great deal of information, amending direct-approach environmental studies (Chong et al. 2013). They create the opportunity of deep genetic analysis as well as investigation of physiological traits. In this regard, we investigated the genetic and metabolic characteristics of 43 *Polaromonas* isolates originating from two Arctic and one Antarctic glacier to assess the variability of this genus on a local and a global scale. We hypothesize that isolates from neighboring glaciers will display similar characteristics, leading to a clear distinction between northern and southern polar region originating strains. To test this hypothesis, we used 16S rRNA gene sequences along with the intergenic transcribed spacers (ITS) to discriminate closely related strains. Phenotypic microarray technology was employed to further diverge the isolates based on their metabolic profiles. Obtained data served to create dendrograms, highlighting the variability within those strains. This approach has not been applied to *Polaromonas* isolates before, therefore providing valuable data for environmental microbiology.

## Materials and methods

### Sites and sampling

Hans and Werenskiold Glaciers are located on the north shore of the Hornsund Fiord at Spitsbergen Island (Svalbard Archipelago) in Arctic. Hans Glacier, a grounded tidewater glacier has a surface of about 57 km^2^ and its bottom reaches 100 m below sea level. Maximum ice thickness was estimated to be 400 m. Werenskiold Glacier is a land-based valley glacier next to Hans Glacier (Grzesiak et al. 2015b). Ecology Glacier is situated at the western shore of Admiralty Bay, on King George Island, South Shetland Archipelago, Antarctica (Grzesiak et al. 2015a).

Ice and cryoconite material were taken from 3 points on the glaciers surface in a transect running up the glacier, from glacial terminus area to the snow line at the top of the ablation zone. The transect on Hans Glacier had a length of 5120 m, on Werenskiold Glacier—3420 m and on Ecology Glacier—1841 m.

Ice from the glacier’s surface (approx. 20 cm) was crushed with an 70 % ethanol solution sterilized and deionized water-washed Tonar ice auger (158 cm long, 130 mm diameter), collected using sterile plastic spatulas and placed into sterile plastic bags. The crushed ice was gathered from 5 points per sampling site, in an area of 100 m^2^. Pooled cores totaled 3 kg per site. Cryoconite holes were drained of water and sediment with a 160 mL sterile plastic syringe, and the material was transported in 500 mL sterile bottles to a field laboratory and processed within 2 h. Five cryoconite holes per site were drained and pooled.

### Sample preparation and strain isolation

Ice samples were melt in a refrigerator (4 °C) before processing for microbiological analyses. Cryoconite material was shaken gently on a universal shaker (Premed, model 327) (120 rpm, 20 min, 5 °C). Suspensions were then returned to the refrigerator for 10–20 min to allow larger particles to settle. Aliquots of 1, 0.5 and 0.1 mL were spread-plated on R2A agar plates (Biocorp) and incubated in darkness at 4 °C for 6 weeks. After 6 weeks of incubation, several colony types that differed in terms of size, color, shape, and other colony characteristics were selected per sample. Pure isolates derived from streaking colonies for isolation were passed through the nonstaining (KOH) test for determination of Gram reaction (Buck 1982). Only those isolates that exhibited the reaction characteristic to Gram-negative bacteria were kept for further investigation.

### Strain DNA isolation, 16S rRNA region and ITS amplification and sequencing

DNA isolation from bacterial isolates for PCR was carried out using the boiling lysis method. *Polaromonas* cells were relatively easy to open using this simple method. Single colony was picked from R2A agar plate and suspended in 50 μl of sterile MiliQ water. The suspension was boiled in 98 °C for 2 min and cooled to 8 °C in thermal cycler following centrifugation for 1 min in microcentrifuge.

One µL of supernatant was used for PCR. Amplification targeted two regions: (I) 16S rRNA gene, using universal primers 27F and 1492R (Lane 1991) and (II) the internal transcribed spacer (ITS) between 16S rRNA gene and 23S rRNA gene region with use of the primers 1407F, targeting inside the 16S rRNA gene gene and 242R, targeting inside the 23S rRNA gene gene (Lane 1991). Both PCR amplifications were done in the same reaction conditions: 1 min of 95 °C initial denaturation followed by 30 cycles of 95 °C for 15 s, 55 °C annealing for 15 s and elongation 72 °C for 1 min, using DreamTaq polymerase (Thermo Scientific—Fermentas). Obtained PCR products (~1500 bp for 16S rRNA gene fragment and ~1100 bp for ITS fragment) were purified using Exonuclease I/Alkaline phosphatase mix (Thermo Scientific—Fermentas). 16S rRNA gene amplicons were sequenced using internal 16S rRNA gene primers: 341F, 518R and 928F (Weidner et al. 1996) and ITS PCR products were sequenced with PCR primers with use of BigDye Terminator v.3.1 chemistry and ABI3730xl genetic analyzer at the DNA Sequencing Laboratory (Institute of Biochemistry and Biophysics PAS). Sequencing reads were manually corrected and assembled into contigs using Seqman software (DNAStar).

### Microarray metabolic fingerprinting

The selected strains were cultivated in R2A broth on a rotary shaker (WL-972, JWElectronics) for 7 days in 10 °C. After the given incubation time, the cells were harvested by centrifugation in a sterile 2 mL Eppendorf-type tube (9000 rpm for 3 min in a MPW-52 microcentrifuge), washed twice and suspended in sterile 0.9 % saline. Bacterial suspensions were added to a vial of GEN III MicroPlate IF C inoculation fluid until transmittance reached 90 %. 100 μL aliquots of each suspension were added to each well of Biolog GEN III microplates (Biolog Inc., Hayward, CA, USA). The plates were incubated in darkness at 10 °C, the color development was measured at 590 nm with a microplate reader (OmniLog) and cellular respiration was measured kinetically by determining the colorimetric reduction of tetrazolium dye. Data were collected approximately three times a week over a 21 day period. The GEN III MicroPlates assesses the ability of a broad range of bacteria to utilize a pallet of different carbohydrates, amino and carboxylic acids, respiration in varying salinity and pH conditions as well as in the presence of various growth inhibitors (+one control well with no-carbon and one positive control). Absorbance data from the different reading times (given in OmniLog arbitrary units) were first blanked against the time “zero” reading and then the values were blanked against the respective control well containing no-carbon source. Ability to respire in given conditions was scored as positive when it was ≥30 % of the respective positive control well value.

### Sequence identification, whole genome sequencing and dendrogram construction

16S rRNA gene fragments and ITS nucleotide sequences were aligned against reference sequence database GenBank (Johnson et al. 2008) using BLAST (Altschul et al. 1990) and using the RDP classifier online program (Cole et al. 2009). Sequences scoring in both databases as *Polaromonas* sp. were taken for further processing.

The ITS region sequence was used to discriminate closely related strains, in total 43 isolates were typed and submitted for whole genome sequencing using Illumina technology. For this purpose, total DNA was isolated from 50 ml R2 broth cultures using CTAB/lysozyme method (Wilson 1987). DNA quality was checked on agarose gel and template quantity was estimated using fluorometry by Qubit 2.0 fluorimeter. Illumina shotgun library was constructed using KAPA reagents. Sequencing was done in paired end mode on the MiSeq sequencer using 600 cycle chemistry kit. Obtained sequence reads were trimmed by quality using FastX toolkit and assembled into contigs using Newbler de novo assembler v3.0 (Roche).

Whole genome sequencing data of *Polaromonas* isolates was used to obtain complete sequence of 16S rRNA gene fragment and ITS regions. Bacterial rRNA gene region was extracted from the assembly dataset based on analysis of Newblers ContigGraph.txt result file. Contigs of the size c.a. 5.6 kb with 5–6 times higher sequence coverage than the rest of the assembly were taken for analysis. 16S rRNA gene fragment and ITS isolate sequence was aligned to 5.6 kb sequence region and manually inspected using Seqman (DNAStar) program. As a result, complete nucleotide sequences of these two regions were extracted based on 16S rRNA gene fragment and ITS PCR primers positions. 16S rRNA gene and ITS sequences were deposited in GenBank under accession numbers KU586628-KU586713.

Multiple sequence alignments were performed using ClustalW program. Phylogenetic trees were constructed using MEGA6 software and Neighbour-Joining method. Bootstrap values for phylogenetic comparisons were based on 1000 pseudoreplicates. Isolate designations indicate the hemisphere and glacier of origin: N—Arctic, S—Antarctica, H—Hans Glacier, W—Werenskiold Glacier, E—Ecology Glacier. 16S and ITS sequences of *P*. *naphthalenivorans* strain CJ2 and *Polaromonas* sp. strain JS666 were used as reference due to the complete gene sequences. *Rhodoferax ferrireducens* strain T118 sequences were used as outgroups.

Clustering analysis of the *Polaromonas* strains responses in GEN III microplates was performed using the unweighted pair-group method and the Euclidean distance (UPGMA) for dendrogram construction. Data were analyzed statistically using Statistica version 10 (StatSoft Inc.) and Canoco ver. 4.5 for Windows (Ter Braak and Šmilauer 2002) for the principal component analysis. PCA was conducted using metabolic and sequence-based molecular data. For the latter a percentage of dissimilarity between a given sequence and the respective reference sequence of *R. ferrireducens* was calculated.

## Results

Forty-three *Polaromonas* isolates were obtained from surface of Arctic and Antarctic glaciers. Nineteen isolates came from Ecology Glacier surface (King George Island, Antarctica), 12 from Hans Glacier and 12 from Werenskiold Glacier (each located at Spitsbergen Island, Arctic). Blastn searches indicate 99 % similarity of the Antarctic strains to *Polaromonas vacuolata*, whereas Arctic strains showed closest similarity to *P. naphthalenivorans*, *P. cryoconiti* and *P. glacialis*.

**Fig. 1:**
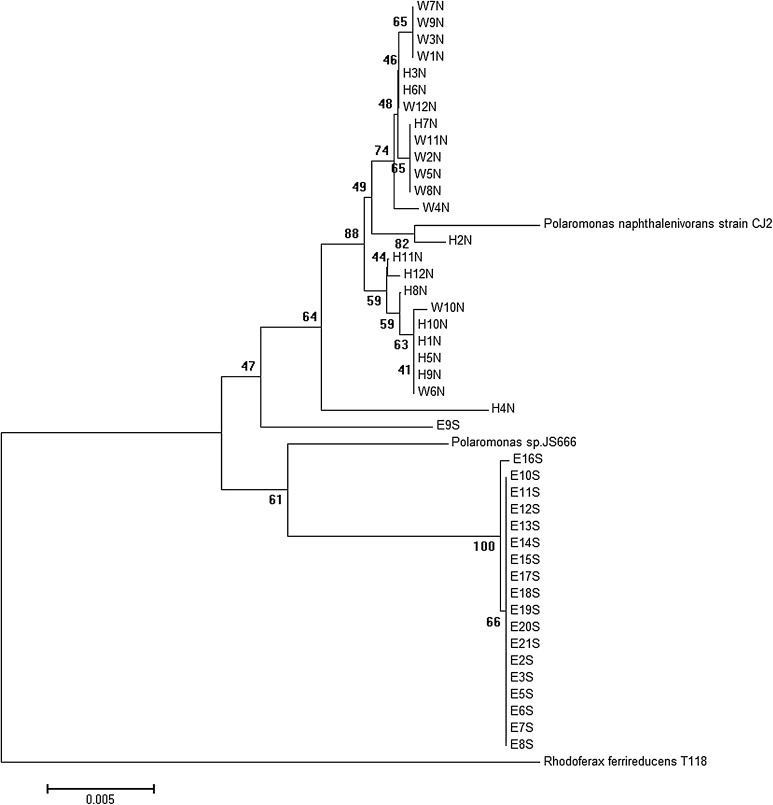
Phylogenetic tree constructed using complete 16S rRNA gene sequences of *Polaromonas* isolates. Isolate designations indicate the hemisphere and glacier of origin (*N* Arctic, *S* Antarctica, *H* Hans Glacier, *W* Werenskiold Glacier, *E* Ecology Glacier). The tree was built with the neighbor-joining method. Bootstrap values are indicated at the nodes. *R. ferrireducens* strain T118 sequence has been used as an outgroup

The phylogenetic tree based on complete 16S rRNA gene sequences is shown in Fig. 1. Three groups emerged when clustering the sequences. The Ecology Glacier strains form a uniform group, distant from the Arctic isolates, which clustered in two subgroups, comprising roughly the strains from a particular glacier, although with some isolates from the other glacier mixing in. The Antarctic strain E9S clustered loosely with the Arctic strains.

**Fig. 2:**
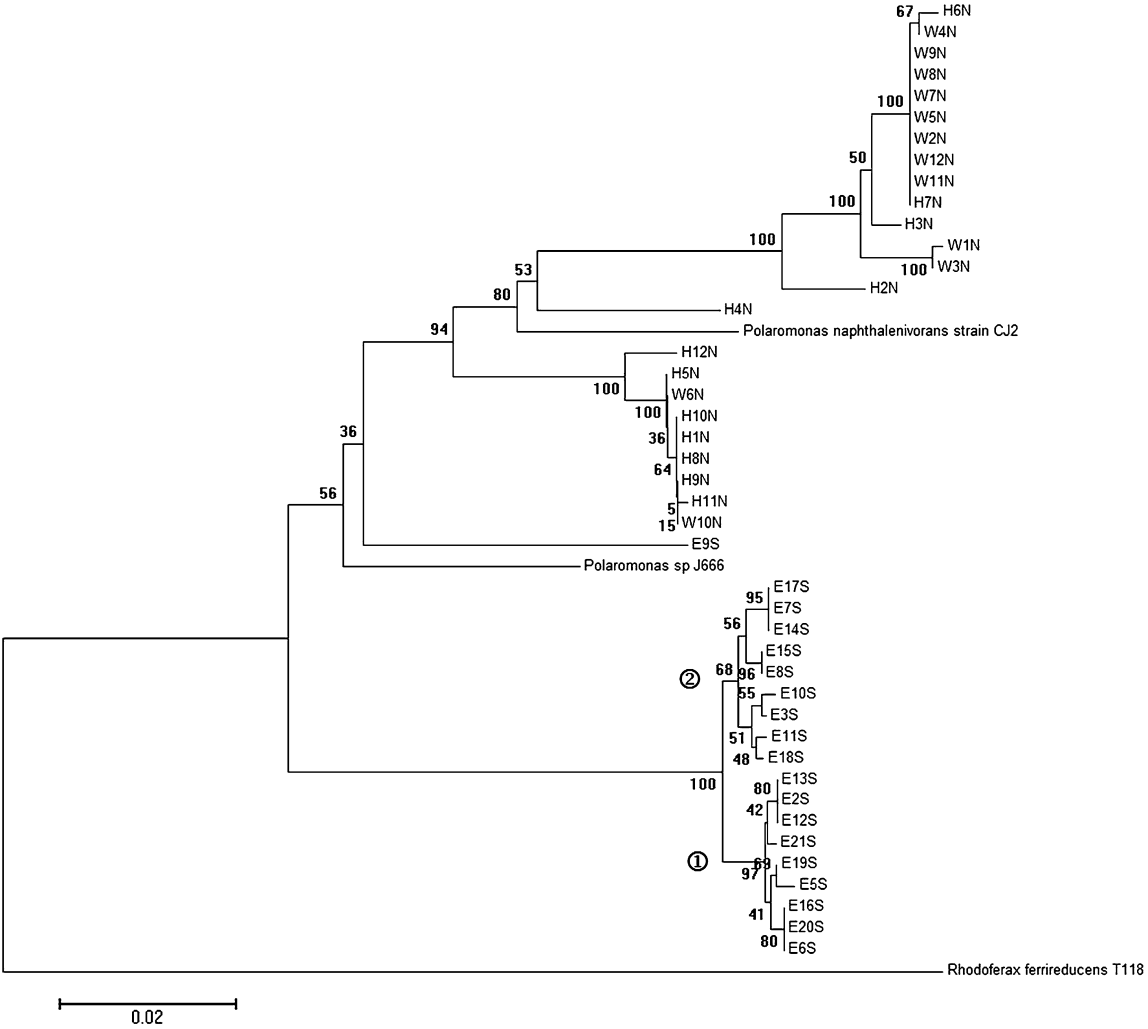
Phylogenetic tree constructed using complete ITS sequences of *Polaromonas* isolates. Isolate designations indicate the hemisphere and glacier of origin (*N* Arctic, *S* Antarctica, *H* Hans Glacier, *W* Werenskiold Glacier, *E* Ecology Glacier). The tree was built with the neighbour-joining method. Bootstrap values are indicated at the nodes. *R. ferrireducens* strain T118 sequence has been used as outgroups. ➀ ITS Ecology Glacier group 1; ➁ ITS Ecology Glacier group 2

The phylogenetic tree based on internal transcribed spacer (ITS) sequences is shown in Fig. 2. The Antarctic isolates are more differentiated. Two groups emerged within the Ecology Glacier isolated strains, the two major clusters of Arctic isolates remained, with the Antarctic isolate E9S clustering again with the Arctic group.

The percentage of strains displaying positive responses in the GENIII plates is displayed in Table 1 and Table 1S. All examined strains efficiently reduced tetrazolium violet (positive response) in pH 6, with l-lactic acid as a carbon source and in the presence of 1 % sodium lactate. Other frequently utilized carbon sources were: glycerol, l-alanine, l-aspartic acid, l-glutamic acid, d,l-malic acid and β-hydroxy-d,l-butyric acid. Major differences between the isolates from particular glaciers have emerged when examining responses at pH5, α-d-glucose, d-galacturonic acid, l-galactonic acid lactone, d-gluconic acid, γ-amino-butyric acid and acetic acid. 11 % of Antarctic strains respired in pH5, whereas over 50 % of Arctic strains gave a positive response at this pH level. Carbohydrates were on average weakly assimilated, with the exception of α-d-glucose which was utilized by 47 % of the Ecology Glacier strains, but none of the Arctic glacier strains. Gluconic acid was assimilated by the majority of the Arctic strains and none on the Antarctic strains. Acetic acid utilization has been confirmed in a great majority of the Hans and Werenskiold Glaciers strains, yet only in 26 % of the Ecology Glacier strains.

**Table 1:** Percentage of positive responses of *Polaromonas* isolates in selected wells showing differences and similarities between glaciers

Metabolic trait	Glacier			Metabolic trait	Glacier		
	E	H	W		E	H	W
ph6	100	100	100	Vancomycin	58	67	67
ph5	11	50	75	Tetrazolium violet	11	0	0
1 % NaCl	100	92	100	Tetrazolium blue	5	58	33
α-d-Glucose	47	0	0	p-Hydroxy-phenylacetic acid	0	17	8
d-Fucose	5	0	0	Methyl pyruvate	42	42	75
1 % sodium lactate	100	100	100	l-Lactic acid	100	100	100
d-Mannitol	32	0	0	α-Keto-glutaric acid	42	42	67
d-Arabitol	32	0	0	d-Malic acid	74	42	100
Glycerol	63	25	83	l-Malic acid	68	92	100
Rifamycin SV	95	100	100	Bromo-succinic acid	37	25	75
l-Alanine	84	67	92	Lithium chloride	11	0	0
l-Aspartic acid	95	100	100	Tween 40	37	75	42
l-Glutamic acid	89	92	100	γ-Amino-butyric acid	5	42	83
l-Pyroglutamic acid	37	8	0	α-Hydroxy-butyric acid	42	33	58
Lincomycin	100	67	100	β-Hydroxy-d, l-Butyric acid	89	100	100
d-Galacturonic acid	5	33	83	α-Keto-butyric acid	32	25	8
l-Galactonic acid lactone	5	8	75	Acetoacetic acid	47	67	17
d-Gluconic acid	0	67	100	Propionic acid	11	17	50
d-Glucuronic acid	26	33	58	Acetic acid	26	100	92
Quinic acid	5	17	0	Aztreonam	63	50	42
d-Saccharic acid	0	8	33	Sodium butyrate	0	33	25

*H* Hans Glacier, *W* Werenskiold Glacier, *E* Ecology Glacier

**Fig. 3:**
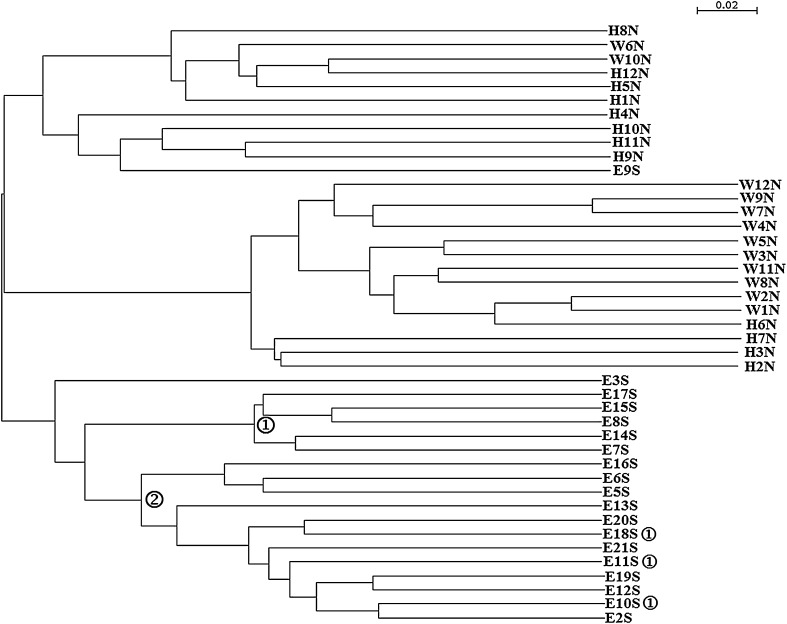
Dendrogram constructed using data obtained from GENIII microplate metabolic features of *Polaromonas* isolates. Isolate designations indicate the hemisphere and glacier of origin (*N* Arctic, *S* Antarctica, *H* Hans Glacier, *W* Werenskiold Glacier, *E* Ecology Glacier). ➀ ITS Ecology Glacier group 1; ➁ ITS Ecology Glacier group 2

The tree based on metabolic traits of the strains obtained by means of GenIII Biolog Plates is shown in Fig. 3. Three distinct groups have emerged, similarly as in the phylogenetic tree based on complete 16S rRNA gene sequences. Within the Antarctic isolate group, two subgroups have formed similarly as in the ITS tree, although with some exceptions as indicated by the circled numerals.

**Fig. 4:**
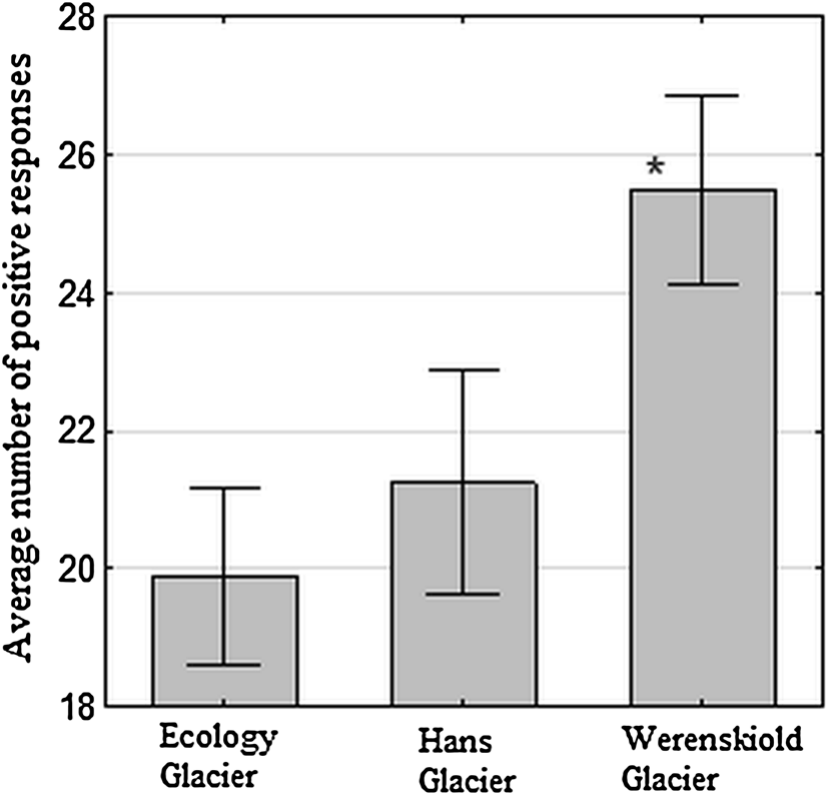
Number of positive reactions in GENIII microplates of *Polaromonas* isolates from a particular glacier. *Asterisk* indicates a statistical significance of *p* < 0.05

The average positive response numbers did not differ much between the Glaciers, with Ecology Glacier displaying 19.9 positive responses, Hans Glacier isolates 21.2 and Werenskiold Glacier isolates 25.5 (Fig. 4). Yet, the difference between Ecology and Werenskiold Glacier isolates response numbers was statistically significant (*p* < 0.05).

**Fig. 5:**
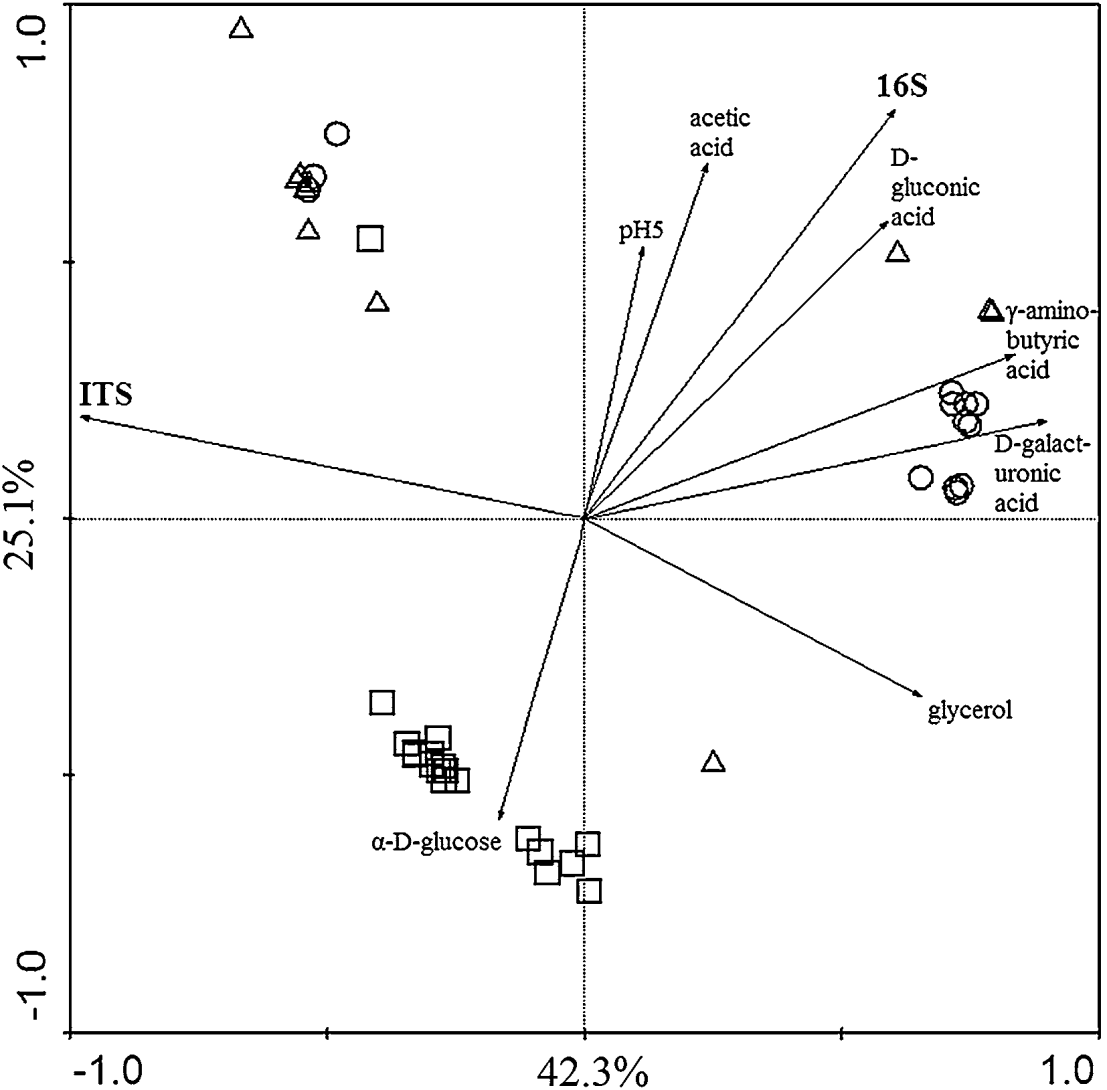
Principal component analysis clustering of isolates based on 16S rRNA gene, ITS sequences and metabolic fingerprinting. *Square* Ecology Glacier isolates, *Triangle* Hans Glacier isolates, *Circle* Werenskiold Glacier isolates

The principal component analysis clustered the *Polaromonas* strains in 3 groups, according to the glaciers which they were isolated from, with intermixing of single strains. Characteristics like 16S rRNA gene sequences, assimilation of acetate, gluconate and glucose differentiated the Arctic from the Antarctic strains. ITS sequences, ability to oxidize glycerol, d-galacturonic acid and γ-amino-butyric acid differentiated arctic isolates into 2 groups (Fig. 5).

## Discussion

Members of the *Polaromonas* genus were isolated from a variety of habitats, were reported via direct diversity assessment methods from various ecosystems and were considered as a marker when investigating polar, marine and freshwater environments (Willems 2014). In this study, we present the genetic and metabolic variability within *Polaromonas* isolates obtained from supraglacial habitats, such as surface ice and cryoconite hole sediment from two Arctic and one Antarctic glacier.

### Biogeography, selection and adaptation

Phylogenetic clustering of 16S rRNA gene sequences of investigated *Polaromonas* spp. differentiated them into 2 distinct groups—the Arctic and the Antarctic. 16S rRNA gene sequence difference between those two clades was 2.7 % on average, which designates the Antarctic and the Arctic isolates as different species (Jogler et al. 2011). A similar situation was observed by Cameron et al. (2012), when examining bacterial communities in cryoconite holes of Arctic and Antarctic glaciers by means of T-RFLP. Dispersal of psychrophilic bacteria between poles was discussed before (Staley and Gosink 1999). Migrating birds, cold, deep sea currents and upper atmosphere air masses were taken into consideration as possible transport agents of polar microbiota. They were all dismissed due to high-temperature amplitudes, slow rate or simply because of lack of evidence for their occurrence. However, Sattler et al. (2001) showed in following years, that bacterial cells can survive and even reproduce in supercooled cloud droplets at high altitudes. In this respect, *Polaromonas* sp. DNA sequences were recovered from air sampled at Fløyen Mountain, Norway (Fahlgren et al. 2010). Whether those cells are capable of withstanding a journey from one pole to another remains unclear.

The Arctic cluster was separated into two groups, each of them containing a majority of isolates from one glacier clustered together with a few isolates from the neighboring glacier. Such “overlapping” of bacterial sequences was also recognized when analyzing community structures of 3 adjacent Svalbard glaciers (Edwards et al. 2013). The composition of the two groups, emerging on the 16S rRNA gene tree within the Arctic clade, remained the same on the ITS tree. Furthermore, the groups were maintained when constructing the tree based on phenotypic traits. This implies that isolates within those two groups diverged genetically and metabolically from one another a considerable time in the past. Whether the dominance of one group on a glacier is due to selection pressure or simply by the proximity of the glacier to the groups primary reservoir remains unclear. However, profound differences in physico-chemical values of glacial surfaces not only between two hemispheres but also within one region were reported previously (Anesio et al. 2009; Grzesiak et al. 2015b; Cameron et al. 2012), which implies, that selection forces are of different quality on each glacier. Xiang et al. (2009) postulated that the shape of microbial communities on glacier is caused by the selection of deposited microbial cells. Investigations on the Ecology Glacier (Grzesiak et al. 2015a) and on the Werenskiold and Hans glaciers (Grzesiak et al. 2015b) regarding physico-chemical controls of microbial supraglacial communities may shed some light on the *Polaromonas* isolates’ metabolic traits as adaptations to conditions on a particular glacier. Most of the *Polaromonas* strains from Arctic glaciers were active in pH 5, whereas only 11 % of the Antarctic strains could respire in such conditions. This could be explained by the pH of the glacier surface, where ice and cryoconites of Hans and Werenskiold Glaciers had a pH ranging from 3.34 to 4.77 (Grzesiak et al. 2015b), the Ecology Glacier surface displayed pH in the range of 6.10–7.15 (Grzesiak et al. 2015a). The lack of activity of investigated *Polaromonas* cells at salt concentrations 4 % and above can also be explained by supraglacial conditions. Dissolved salts amounts in Ecology Glacier cryoconite hole water were minimal, as indicated by its low conductivity, not exceeding 3.4 μS cm^−1^ (Mieczan et al. 2013). *Polaromonas vacuolata*, isolated from Antarctic sea ice, is the only halophile within this genus described to date, although its salinity range of growth is quite high (0–6 %) (Irgens et al. 1996). Considering marine aerosol dispersal by high velocity Antarctic winds (Pearce et al. 2009), supraglacial strains obtained in this study, closely related to *P. vacuolata* may be of sea origin.

High affinity of investigated *Polaromonas* strains for simple organic acids, hints an abundance of those carbon compounds in the supraglacial environment. Sources of those acids may be diverse, allochtonic, as well as autochtonic (Stibal et al. 2008). It has been proven on several occasions that in many aquatic environments organic acids are a result of photodegradation of recalcitrant substances, like humic and fulvic acids (Wetzel et al. 1995; Watanabe et al. 2009). They can also be produced on the glacier surface via microbes, leaching from live cells during stress conditions (Medina-Sánchez and Villar-Argaiz 2006) or being actively released to solubilize phosphate and other biogenic elements (Rodríguez and Fraga 1999). Scavenging of those small molecules may give the *Polaromonas* an advantage in those oligotrophic conditions.

### Microevolution, phenotype and niche separation

**Fig. 6:**
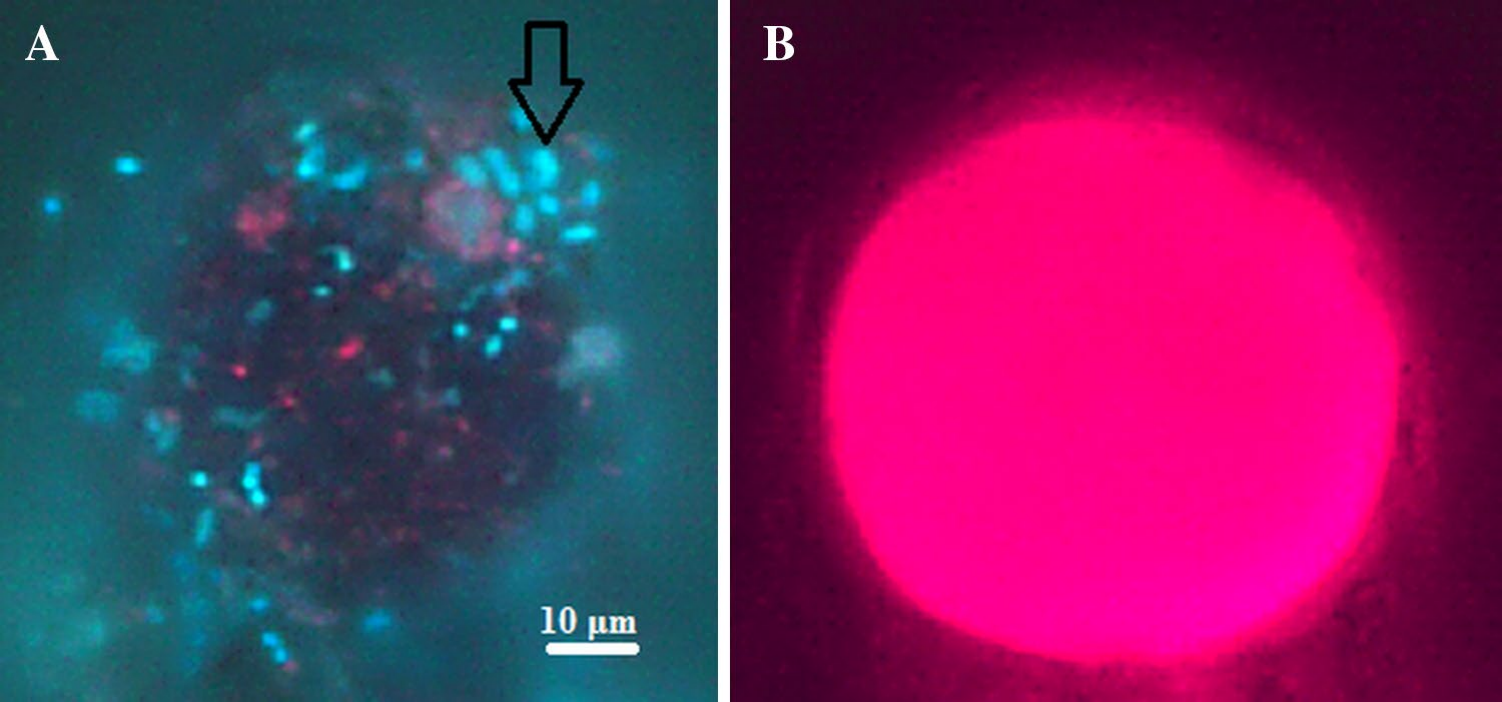
Microphotograph of algae and bacteria agglomerate from Ecology Glacier surface: **a** DAPI stained bacterial cells (*black arrow*) imbedded in extracellular matrix of a round algae cell (under UV light). **b** Autofluorescence of the same algae cell under *green*-light excitation

The isolates comprising the Antarctic branch of the 16S rRNA gene tree were separated into two branches on the ITS tree. As the ITS sequences evolve more quickly than the 16S rRNA gene sequences it is safe to assume that the divergence within this group has occurred fairly recently, perhaps even on the glacier itself, following deposition and selection. Some isolates still share properties not present in their closest genetic relatives. This could further support the recent divergence of those two groups. Differences in ITS sequences and phenotypes among bacteria sharing the same 16S rRNA gene sequence were observed before (Jaspers and Overmann 2004; Brown et al. 2005) and were repeatedly linked to niche separation (Brown and Fuhrman 2005; Hahn and Pöckl 2005; Jogler et al. 2011). Those authors pointed spatial, temporal, temperature and dissolved organic carbon quality-dependent ecological niche differentiation. Spatio-temporal causes seem not to apply to the diversification observed in this study, as the sampling on the Ecology Glacier surface was undertaken within a few hours and the clusters contain isolates from several sampling points. Chemical composition within the particular site or even biological interactions might be therefore responsible. Several features of the Antarctic *Polaromonas* strains points towards host–symbiont interactions as possible drivers of such diversification. Nearly half of the strains from Ecology Glacier surface exhibited d-glucose utilization capability, but none of those strains respired when gluconic acid was the only carbon source, which was readily consumed by Arctic strains. In the heavily solar radiation impacted glacial surfaces (Säwström et al. 2002) glucose may easily be oxidized inter alia to gluconic acid (Phillips and Rickards 1969). The phenomenon that the Antarctic strains utilize glucose yet lack the ability to degrade more complex carbohydrates like dextrin may suggest that they tightly adhere to algae cells, which secrete simple carbohydrates in stress conditions that are immediately consumed by bacteria, before being oxidized. Microscope observations of aggregates containing algae and bacteria were confirmed from Ecology Glacier surface (Fig. 6). Such host–symbiont interaction may explain the separation within the Ecology Glacier isolates, where the niche separations occur in accordance with host specificity. Such phenomenon has been observed by Šimek et al. (2011), where *Limnohabitans* sp. strains were proposed to respond to different extracellular algae-derived substances by niche separation. Antarctic *Polaromonas* strains also have on average lower assimilation capabilities than Arctic strains. Loss of function in symbionts were connected with growing dependency to host metabolites (Ochman and Moran 2001). Furthermore, *Polaromonas*-like bacterium has been found in a consortium with a phototrophic partner (Kanzler et al. 2005), suggesting that within this genus, symbiotic interaction might be common.

In conclusion, the presented data amend the information on *Polaromonas* spp. biogeography, evolution and physiology. Members of the genus *Polaromonas* occupy supraglacial habitats of Arctic and Antarctic glaciers. Darcy et al. (2011) postulate a global dispersal of most *Polaromonas* phylotypes. Distribution by air currents is likely the way of propagating the cells of this genus, but the efficiency of seeding a glacial surface with cells that were transported across great distances and from a variety of different environments might be greater on local than global scales. Selection mechanisms caused by prevailing environmental conditions on a glacier may drastically reduce the deposited diversity. Furthermore, biotic and abiotic factors may drive postselectional niche separation and microevolution within the *Polaromonas* genus.

## Electronic supplementary material

Below is the link to the electronic supplementary material.

Supplementary material 1 (DOCX 21 kb)
