# Supplementary material for: Evidence of adaptation, niche separation and microevolution within the genus Polaromonas on Arctic and Antarctic glacial surfaces
Source: Extremophiles. 2016 Apr 20;20:403–13. doi: 10.1007/s00792-016-0831-0 (PMC4921121; doi:10.1007/s00792-016-0831-0)
Supplement: Supplementary file 1 — Supplementary material 1 (DOCX 21 kb) [file 792_2016_831_MOESM1_ESM.docx]

| **Negative Control**   \| **E 0%** \| \| --- \| \| **H 0%** \| \| **W 0%** \| | **Dextrin**   \| **E 0%** \| \| --- \| \| **H 0%** \| \| **W 0%** \| | **D-Maltose**   \| **E 0%** \| \| --- \| \| **H 0%** \| \| **W 0%** \| | **D-Trehalose**   \| **E 0%** \| \| --- \| \| **H 0%** \| \| **W 0%** \| | **D-Cellobiose**   \| **E 0%** \| \| --- \| \| **H 0%** \| \| **W 0%** \| | **Gentiobiose**   \| **E 0%** \| \| --- \| \| **H 0%** \| \| **W 0%** \| | **Sucrose**   \| **E 0%** \| \| --- \| \| **H 0%** \| \| **W 0%** \| | **D-Turanose**   \| **E 0%** \| \| --- \| \| **H 0%** \| \| **W 0%** \| | **Stachyose**   \| **E 0%** \| \| --- \| \| **H 0%** \| \| **W 0%** \| | **Positive Control**   \| **E 100%** \| \| --- \| \| **H 100%** \| \| **W 100%** \| | **pH 6**   \| **E 100%** \| \| --- \| \| **H 100%** \| \| **W 100%** \| | **pH 5**   \| **E 11%** \| \| --- \| \| **H 50%** \| \| **W 75%** \| |
| --- | --- | --- | --- | --- | --- | --- | --- | --- | --- | --- | --- | --- | --- | --- | --- | --- | --- | --- | --- | --- | --- | --- | --- | --- | --- | --- | --- | --- | --- | --- | --- | --- | --- | --- | --- | --- | --- | --- | --- | --- | --- | --- | --- | --- | --- | --- | --- |
| **D-Raffinose**   \| **E 0%** \| \| --- \| \| **H 0%** \| \| **W 0%** \| | **α-D-Lactose**   \| **E 0%** \| \| --- \| \| **H 0%** \| \| **W 0%** \| | **D-Melibiose**   \| **E 0%** \| \| --- \| \| **H 0%** \| \| **W 8%** \| | **β-Methyl-D-Glucose**   \| **E 0%** \| \| --- \| \| **H 0%** \| \| **W 0%** \| | **D-Salicin**   \| **E 0%** \| \| --- \| \| **H 0%** \| \| **W 0%** \| | **N-Acetyl-D-Glucosamine**   \| **E 0%** \| \| --- \| \| **H 0%** \| \| **W 0%** \| | **N-Acetyl-β-D-**  **Mannosamine**   \| **E 0%** \| \| --- \| \| **H 0%** \| \| **W 0%** \| | **N-Acetyl-D-**  **Galactosamine**   \| **E 5%** \| \| --- \| \| **H 0%** \| \| **W 0%** \| | **N-Acetyl**  **Neuraminic Acid**   \| **E 5%** \| \| --- \| \| **H 0%** \| \| **W 0%** \| | **1% NaCl**   \| **E 100%** \| \| --- \| \| **H 92%** \| \| **W 100%** \| | **4% NaCl**   \| **E 5%** \| \| --- \| \| **H 8%** \| \| **W 0%** \| | **8% NaCl**   \| **E 0%** \| \| --- \| \| **H 0%** \| \| **W 0%** \| |
| **α-D-Glucose**   \| **E 47%** \| \| --- \| \| **H 0%** \| \| **W 0%** \| | **D-Mannose**   \| **E 0%** \| \| --- \| \| **H 0%** \| \| **W 0%** \| | **D-Fructose**   \| **E 0%** \| \| --- \| \| **H 0%** \| \| **W 0%** \| | **D-Galactose**   \| **E 0%** \| \| --- \| \| **H 0%** \| \| **W 0%** \| | **3-Methyl Glucose**   \| **E 0%** \| \| --- \| \| **H 0%** \| \| **W 0%** \| | **D-Fucose**   \| **E 5%** \| \| --- \| \| **H 0%** \| \| **W 0%** \| | **L-Fucose**   \| **E 5%** \| \| --- \| \| **H 0%** \| \| **W 0%** \| | **L-Rhamnose**   \| **E 0%** \| \| --- \| \| **H 0%** \| \| **W 0%** \| | **Inosine**   \| **E 0%** \| \| --- \| \| **H 0%** \| \| **W 0%** \| | **1% Sodium**  **Lactate**   \| **E 100%** \| \| --- \| \| **H 100%** \| \| **W 100%** \| | **Fusidic Acid**   \| **E 0%** \| \| --- \| \| **H 0%** \| \| **W 0%** \| | **D-Serine**   \| **E 5%** \| \| --- \| \| **H 0%** \| \| **W 0%** \| |
| **D-Sorbitol**   \| **E 5%** \| \| --- \| \| **H 0%** \| \| **W 0%** \| | **D-Mannitol**   \| **E 32%** \| \| --- \| \| **H 0%** \| \| **W 0%** \| | **D-Arabitol**   \| **E 32%** \| \| --- \| \| **H 0%** \| \| **W 0%** \| | **myo-Inositol**   \| **E 5%** \| \| --- \| \| **H 0%** \| \| **W 0%** \| | **Glycerol**   \| **E 63%** \| \| --- \| \| **H 25%** \| \| **W 83%** \| | **D-Glucose-6-PO4**   \| **E 0%** \| \| --- \| \| **H 0%** \| \| **W 0%** \| | **D-Fructose-6-PO4**   \| **E 0%** \| \| --- \| \| **H 0%** \| \| **W 0%** \| | **D-Aspartic Acid**   \| **E 0%** \| \| --- \| \| **H 0%** \| \| **W 0%** \| | **D-Serine**   \| **E 0%** \| \| --- \| \| **H 0%** \| \| **W 0%** \| | **Troleandomycin**   \| **E 0%** \| \| --- \| \| **H 0%** \| \| **W 0%** \| | **Rifamycin SV**   \| **E 95%** \| \| --- \| \| **H 100%** \| \| **W 100%** \| | **Minocycline**   \| **E 0%** \| \| --- \| \| **H 0%** \| \| **W 0%** \| |
| **Gelatin**   \| **E 0%** \| \| --- \| \| **H 0%** \| \| **W 0%** \| | **Glycyl-L-Proline**   \| **E 5%** \| \| --- \| \| **H 0%** \| \| **W 0%** \| | **L-Alanine**   \| **E 84%** \| \| --- \| \| **H 67%** \| \| **W 92%** \| | **L-Arginine**   \| **E 0%** \| \| --- \| \| **H 0%** \| \| **W 0%** \| | **L-Aspartic Acid**   \| **E 95%** \| \| --- \| \| **H 100%** \| \| **W 100%** \| | **L-Glutamic Acid**   \| **E 89%** \| \| --- \| \| **H 92%** \| \| **W 100%** \| | **L-Histidine**   \| **E 0%** \| \| --- \| \| **H 8%** \| \| **W 0%** \| | **L-Pyroglutamic**  **Acid**   \| **E 37%** \| \| --- \| \| **H 8%** \| \| **W 0%** \| | **L-Serine**   \| **E 0%** \| \| --- \| \| **H 0%** \| \| **W 0%** \| | **Lincomycin**   \| **E 100%** \| \| --- \| \| **H 67%** \| \| **W 100%** \| | **Guanidine HCl**   \| **E 0%** \| \| --- \| \| **H 0%** \| \| **W 0%** \| | **Niaproof 4**   \| **E 5%** \| \| --- \| \| **H 0%** \| \| **W 0%** \| |
| **Pectin**   \| **E 5%** \| \| --- \| \| **H 0%** \| \| **W 0%** \| | **D-Galacturonic Acid**   \| **E 5%** \| \| --- \| \| **H 33%** \| \| **W 83%** \| | **L-Galactonic Acid Lactone**   \| **E 5%** \| \| --- \| \| **H 8%** \| \| **W 75%** \| | **D-Gluconic Acid**   \| **E 0%** \| \| --- \| \| **H 67%** \| \| **W 100%** \| | **D-Glucuronic Acid**   \| **E 26%** \| \| --- \| \| **H 33%** \| \| **W 58%** \| | **Glucuronamide**   \| **E 5%** \| \| --- \| \| **H 0%** \| \| **W 0%** \| | **Mucic Acid**   \| **E 0%** \| \| --- \| \| **H 0%** \| \| **W 0%** \| | **Quinic Acid**   \| **E 5%** \| \| --- \| \| **H 17%** \| \| **W 0%** \| | **D-Saccharic Acid**   \| **E 0%** \| \| --- \| \| **H 8%** \| \| **W 33%** \| | **Vancomycin**   \| **E 58%** \| \| --- \| \| **H 67%** \| \| **W 67%** \| | **Tetrazolium Violet**   \| **E 11%** \| \| --- \| \| **H 0%** \| \| **W 0%** \| | **Tetrazolium Blue**   \| **E 5%** \| \| --- \| \| **H 58%** \| \| **W 33%** \| |
| **p-Hydroxy-Phenylacetic Acid**   \| **E 0%** \| \| --- \| \| **H 17%** \| \| **W 8%** \| | **Methyl Pyruvate**   \| **E 42%** \| \| --- \| \| **H 42%** \| \| **W 75%** \| | **D-Lactic Acid Methyl Ester**   \| **E 0%** \| \| --- \| \| **H 8%** \| \| **W 0%** \| | **L-Lactic Acid**   \| **E 100%** \| \| --- \| \| **H 100%** \| \| **W 100%** \| | **Citric Acid**   \| **E 0%** \| \| --- \| \| **H 0%** \| \| **W 0%** \| | **α-Keto-Glutaric Acid**   \| **E 42%** \| \| --- \| \| **H 42%** \| \| **W 67%** \| | **D-Malic Acid**   \| **E 74%** \| \| --- \| \| **H 42%** \| \| **W 100%** \| | **L-Malic Acid**   \| **E 68%** \| \| --- \| \| **H 92%** \| \| **W 100%** \| | **Bromo-Succinic Acid**   \| **E 37%** \| \| --- \| \| **H 25%** \| \| **W 75%** \| | **Nalidixic Acid**   \| **E 0%** \| \| --- \| \| **H 8%** \| \| **W 0%** \| | **Lithium Chloride**   \| **E 11%** \| \| --- \| \| **H 0%** \| \| **W 0%** \| | **Potassium Tellurite**   \| **E 0%** \| \| --- \| \| **H 0%** \| \| **W 0%** \| |
| **Tween 40**   \| **E 37%** \| \| --- \| \| **H 75%** \| \| **W 42%** \| | **γ-Amino-Butryric Acid**   \| **E 5%** \| \| --- \| \| **H 42%** \| \| **W 83%** \| | **α-Hydroxy-Butyric Acid**   \| **E 42%** \| \| --- \| \| **H 33%** \| \| **W 58%** \| | **β-Hydroxy-D,L-Butyric Acid**   \| **E 89%** \| \| --- \| \| **H 100%** \| \| **W 100%** \| | **α-Keto-Butyric Acid**   \| **E 32%** \| \| --- \| \| **H 25%** \| \| **W 8%** \| | **Acetoacetic Acid**   \| **E 47%** \| \| --- \| \| **H 67%** \| \| **W 17%** \| | **Propionic Acid**   \| **E 11%** \| \| --- \| \| **H 17%** \| \| **W 50%** \| | **Acetic Acid**   \| **E 26%** \| \| --- \| \| **H 100%** \| \| **W 92%** \| | **Formic Acid**   \| **E 0%** \| \| --- \| \| **H 0%** \| \| **W 0%** \| | **Aztreonam**   \| **E 63%** \| \| --- \| \| **H 50%** \| \| **W 42%** \| | **Sodium Butyrate**   \| **E 0%** \| \| --- \| \| **H 33%** \| \| **W 25%** \| | **Sodium Bromate**   \| **E 0%** \| \| --- \| \| **H 0%** \| \| **W 0%** \| |

Tab. 1S. Percentage of *Polaromonas* strains exhibiting positive reactions in wells of a GENIII microplate. H- Hans Glacier isolates, W- Werenskiold Glacier isolates, E – Ecology Glacier isolates.
